# Development and Test of Highly Accurate End Point Free Energy Methods. 4. Expanding Solvents Capability and logBB Prediction

**DOI:** 10.1021/acs.jpcb.6c00810

**Published:** 2026-05-05

**Authors:** Taoyu Niu, Xibing He, Viet Hoang Man, Xujian Wang, Junmei Wang

**Affiliations:** † Department of Pharmaceutical Sciences and Computational Chemical Genomics Screening Center, School of Pharmacy, 6614 University of Pittsburgh, Pittsburgh, Pennsylvania 15261, United States; ‡ Department of Computational and Systems Biology, School of Medicine, University of Pittsburgh, Pittsburgh, Pennsylvania 15261, United States

## Abstract

We reported an expanded Poisson–Boltzmann surface area (PBSA) solvation model for the high-throughput calculation of solvation free energies (SFEs) and partition coefficients (logP) across diverse organic solvents. Furthermore, a predictive model for blood–brain barrier (BBB) permeability (logBB) was developed. Using 1246 experimental SFE measurements from the Minnesota Solvation (MNSol) database, we parametrized the solvent accessible surface area (SASA) nonpolar term for 27 organic solvents, enabling PBSA calculations in a broad set of solvent environments. Across the MNSol data set, PBSA achieves an overall root-mean-square error (RMSE) of 1.10 kcal/mol, compared with 0.98 kcal/mol from quantum mechanical SMD calculations at the B3LYP/6-31G* level. To assess parameter transferability, we computed water-organic solvent logP values for 248 compounds spanning five solvent systems; PBSA yields an overall RMSE of 2.02 log units, slightly higher than the SMD result of 1.5 log units. Finally, we developed a quantitative model for BBB permeability (logBB) using multivariable linear regression based on PBSA-derived hydration free energies and selected organic-solvent SFEs. The model shows stable predictive performance on both the training (*N* = 865) and test (*N* = 97) sets, with RMSE ≈ 0.65 log units and MAE ≈ 0.5 log units, and demonstrates improved rank ordering on the test set (Kendall’s tau = 0.45). Overall, this work extends PBSA applicability to a wide range of solvents and establishes physically motivated and computationally efficient approaches for predicting some key ADMET descriptors.

## Graphical abstract

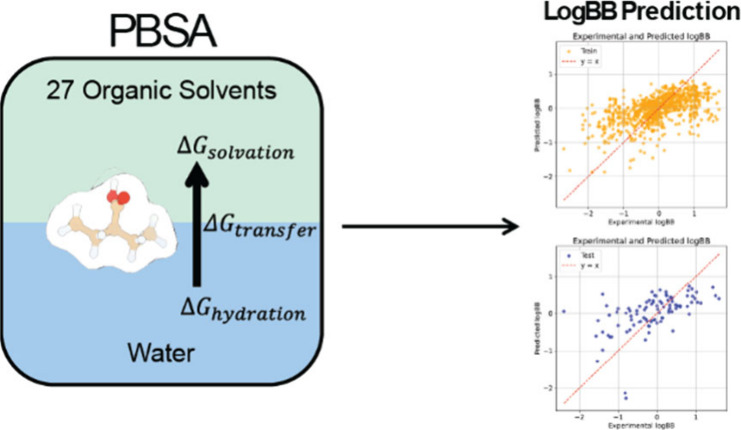

## Introduction

The modeling of solvent effects is essential in electronic structure calculation,^1^ biomolecular electrostatics,^2,3^ and computer-aided drug design. Explicit solvent models can precisely simulate thermodynamic ensembles and enable the analysis of many-body interactions. However, their widespread application is limited by the high computational cost. In contrast, the implicit solvent model approximates solute–solvent electrostatic interactions by treating the solvent as a polarizable dielectric continuum. When combined with appropriate nonpolar models, implicit solvent models provide an efficient framework for calculating solvation free energy (SFE).^4^

SFE is a key physicochemical descriptor in drug discovery. A drug’s aqueous solubility directly influences formulation design and bioavailability, while the partition coefficient (logP) is a central metric for assessing a drug’s absorption, distribution, metabolism, excretion, toxicity (ADMET) properties.^5^ Notably, the solute’s logP from solvent *j* to *i* can be calculated from SFEs:

1 $${\mathrm{logP}}_{i/j}=\frac{-\Delta G_{j\to i}}{RT\;\ln10}$$

2 $$\Delta G_{j\to i}=\Delta G_{i}-\Delta G_{j}$$

A variety of theoretical models have been developed for the calculation of SFEs. The solvation model based on density (SMD) is a representative implicit solvent model that integrates a polarizable continuum model (PCM) with a fine-tuned nonpolar term.^6^ Owing to its design, SMD can be extended to arbitrary solvents once seven solvent-specific parameters are available. Closely related models, including COSMO-RS,^7,8^ the embedded cluster reference interaction site model (EC-RISM),^9^ and the Miertus–Scrocco–Tomasi (MST) model,^10^ have also demonstrated high accuracy in predicting SFEs, as evidenced by their strong performance in SAMPL series blind challenges.^11–19^

Another widely adopted framework for modeling reaction-field electrostatics is based on the Poisson–Boltzmann (PB) equation. PB models offer a favorable balance between accuracy and computational efficiency, making them particularly attractive in drug discovery,^20^ which usually demands intensive calculations and deals with large molecules. The generalized Born (GB) model further improves computational efficiency and enables frequent calculations in molecular dynamics (MD) simulations, although its formulation is generally not as rigorous as that of PB.^21^ Considerable efforts have therefore been devoted to the development and refinement of PB-based solvation models, including advances in numerical PB solvers^22–25^ and calculation protocols. For example, Swanson and co-workers proposed two distinct sets of atomic radii optimized for abrupt and smoothed dielectric boundary,^26^ Venken et al. proposed a heterogeneous dielectric constants scheme in PB for binding affinity calculation,^27^ and a systematical study of PB models for protein–ligand binding affinity calculation has been summarized.^20^ More recently, we developed a set of GAFF2 atom-type-based radii for PBSA by fitting experimental hydration free energy (HFE)^28^ and its application on logP and logD calculations demonstrated outstanding accuracy.^29,30^

The development of deep neural networks has enabled the modeling of solvent effects. Graph neural networks (GNN) have been employed to learn solvent potential of mean force (PMF) from calculated data by either explicit or implicit solvent, with Chen et al. and Airas et al. developing implicit solvent models for MD simulations based on SchNet architecture.^31,32^ More recently, Wu et al. introduced a GNN framework, PBGNN, for predicting PB energies.^33^

In practical drug design, the prediction of solubility and partition coefficients requires high-throughput calculations across multiple solvents. *Ab initio* approaches such as SMD and COSMO-RS are generally too computationally demanding for such applications, while neural-network-based solvent models are often constrained by limited training data and face challenges in achieving broad solvent transferability. In this work, we report parameter sets for 27 organic solvents for the Poisson–Boltzmann Surface Area (PBSA) model, enabling efficient calculations of SFEs and partition coefficients in diverse organic solvents.

Additionally, we developed a predictive model for blood–brain barrier (BBB) permeability (logBB) using multivariable linear regression on PBSA-calculated SFEs, by assuming that logBB depends linearly on SFEs across multiple solvents. LogBB is a key metric for central nervous system (CNS) drug discovery, as it reflects a compound’s ability to reach target sites within the human CNS.^34^ Recently, logBB prediction models have largely been developed using QSAR and machine-learning approaches for either classification or quantification.^35–42^ However, limited data availability has meant that quantitative logBB prediction has typically relied on simpler QSAR methods or classical machine-learning algorithms, which are generally more robust on small data sets.^42,43^

## Methods

### Data Preparation and Processing

PBSA nonpolar parameters were developed by fitting to experimental SFEs from the Minnesota Solvation (MNSol) Database (v2012).^44^ In total, 1246 SFE data points spanning 27 solvents were used for parametrization. Solute geometries for PBSA calculations were taken from the MNSol XYZ files and converted to MOL2 format using Schrödinger Maestro (v11.2).^45^ GAFF2 ^46^ atom types and ABCG2 ^47,48^ atomic charges were assigned using the Antechamber^49^ module in AmberTools.^50^

An independent logP data set was collected from Leo et al.^51^ to evaluate the resulting PBSA solvation models. LogP entries annotated with footnotes in the Leo and co-worker’s data set were excluded, as these values were measured under specific or nonstandard experimental conditions. Furthermore, entries with multiple reported values exhibiting discrepancies greater than 0.1 log units were excluded. For cases where multiple values were available but remained within this threshold, the median value was adopted as the representative logP. For this set, 2D structural information was retrieved from PubChem (https://pubchem.ncbi.nlm.nih.gov/), and 3D geometries were generated with Open Babel 3.1.0 ^52^ using the “--gen3d” option and saved in MOL2 format. Only a single conformation per molecule was used. Atom types and partial charge assignments followed the same protocol used for the MNSol compounds.

For logBB prediction model construction, 1000 compounds with quantitatively measured logBB values were initially obtained from Shaker et al.^43^ It should be noted that, in this data set, experimental approaches for measuring logBB vary widely, ranging from in vivo techniques such as radiolabeling to in vitro methods like immobilized artificial membrane assays. While in vivo experiments generally provide the most accurate logBB values, their technical complexity and cost limit data availability. To address this limitation and improve the generalizability of computational models, in vitro logBB data are incorporated into the modeling process. Data entries with molecular weight >600, salts, and multicomponent (multientity) records were removed, yielding 963 curated logBB data points. A randomly selected test set of 97 molecules was held out, and the remaining 866 molecules were used for training. The distributions of molecular weights and logBB values across training and test set molecules were plotted in Figure 1. Molecular structure preparation, atom types, and partial charges assignment for the logBB data set followed the same procedure employed for the logP data set.

**Figure 1:**
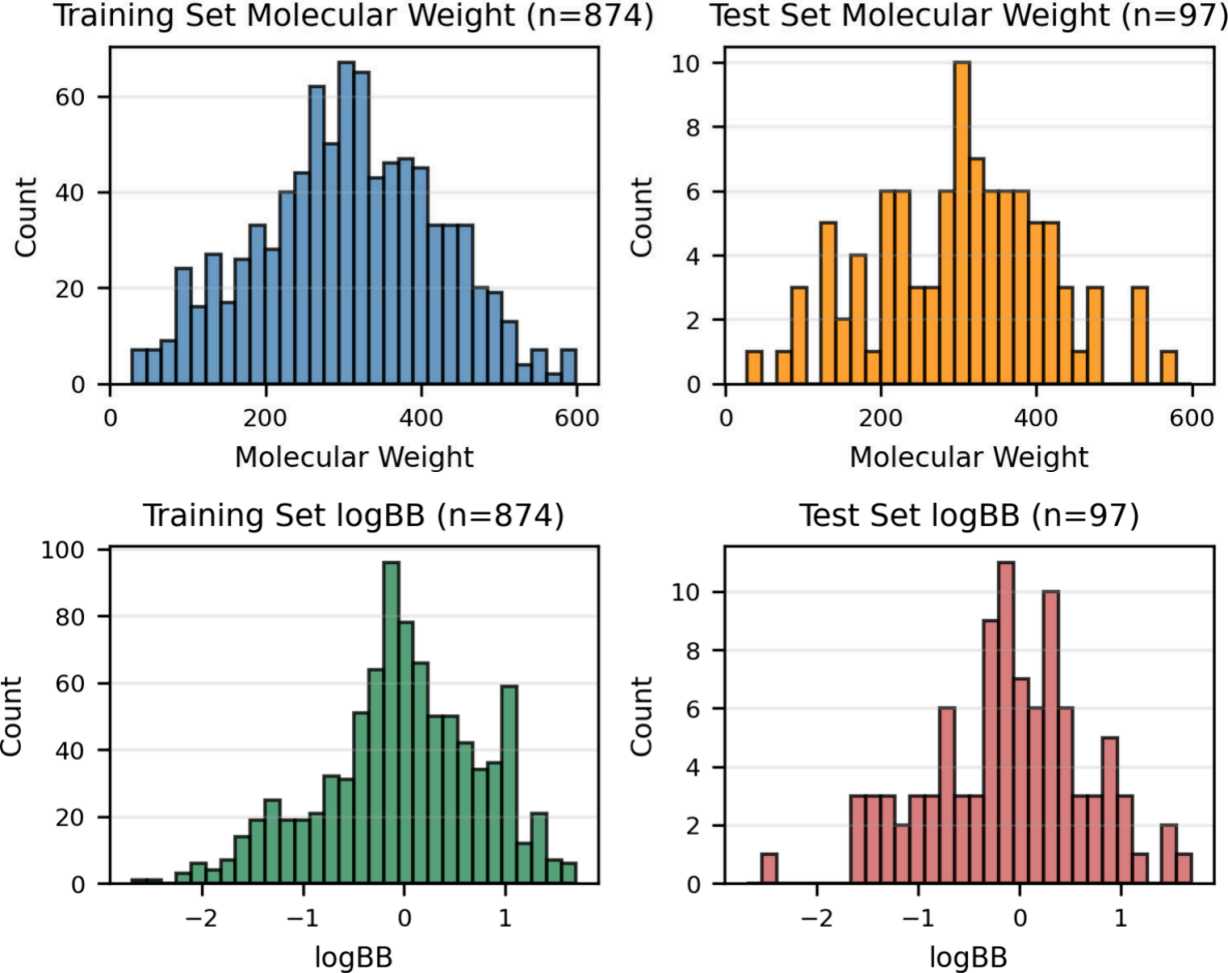
Distribution of molecular weight (Da) and logBB values across the training and test data sets.

## PBSA Calculation and Parametrization

The PB energy was calculated using Delphi V4 release 1.1.^53^ Fine-tuned atomic radii from Sun et al. were used,^28^ and the dielectric boundary was defined as the Coulombic boundary with a probe radius of 1.4 Å. The solvent-accessible surface area (SASA) was computed by using the internal MS program. Additional settings of the PB calculation are the same as in our previous publication.^30^

SFE was calculated as

3 $$G_{solv}=\Delta G_{PB}+\Delta G_{SASA}$$

4 $$\Delta G_{SASA}=\gamma SASA+b$$

where γ and *b* are solvent-specific parameters. In this PBSA model, each solvent is parametrized by three quantities: dielectric constant (ε), γ and *b*. For water, γ = 0.005 and *b* = 1.12 from Sun et al.^28^ LogP was calculated using eq 1.

A systematic search was performed to determine the optimal γ and *b* values for each solvent while keeping the dielectric constant fixed.

## Ab Initio Calculations of Solvation Free Energy

A benchmark calculation using the SMD^6^ implicit-solvent model was performed for SFEs and the test set logP. All calculations were performed using Gaussian 16 ^54^ at B3LYP/6-31G* level of theory. For each solute, the geometry was optimized in solution using a SMD at the same level. The SFE was then obtained as the difference between the SMD energy and the gas-phase single-point energy evaluated with the same geometry.

## Alchemical Calculations of Solvation Free Energy

Another benchmark calculation using thermodynamic integration (TI) was performed for the test set logP values. The alchemical enhanced sampling (ACES) method implemented in AMBER 2022 was used for solvation free energy calculations while GPU-TI was used for hydration free energy calculations.^55^

TLEAP module in AmberTools 2022 ^50^ was used for building the solute–solvent cubic box with a final dimension of ∼30 Å. The simulation protocol is the same as we used in our previous work.^30^ A CPU-TI was performed to equilibrate the systems, and nine λ windows (0.01592, 0.08198, 0.19331, 0.33787, 0.5, 0.66213, 0.80669, 0.91802, and 0.98408) were picked for GPU-TI calculations with the initial configuration sampled from the CPU-TI at λ = 0.01592. The GPU-TI was used for sampling the configurations for ACES simulations. The TI simulations (CPU-TI, GPU-TI and ACES) were performed under periodic boundary conditions and an isothermal–isobaric *NPT* ensemble. System temperature was regulated by Langevin dynamics with a collision frequency of 2.0 ps^–1^. Monte Carlo barostat was used for pressure control, and the SHAKE constraint was turned on for solutes. The last snapshot from GPU-TI at λ = 0.5 served as the initial configuration for ACES with an enlarged solvent box size of ∼46 Å. Nine λ windows (0.1, 0.2, 0.3, 0.4, 0.5, 0.6, 0.7, 0.8, and 0.9) were used for both the solution and gas phases. In total 2 ns simulation was performed for each solute, with the first 0.5 ns simulation was discarded during the free energy calculation.

## Results and Discussion

### PB Solvation Model Development

We first report the fitted PBSA nonpolar parameters for the solvents available in the MNSol database together with error assessments for both PBSA and benchmark SMD calculations (Table 1). Across the full data set, PBSA achieved an overall RMSE of 1.10 kcal/mol for SFEs, compared with that of 0.98 kcal/mol for the SMD benchmark. The PBSA-calculated SFE values for each solute are provided in the . Overall, PBSA is slightly less accurate than SMD for SFE but requires significantly less elapsed time. It is also worth noting that the experimental SFE values used here served not only for PBSA parametrization but also in the parametrization of the SMD model. In our previous work, we investigated the conformational dependence of PBSA accuracy and showed that using a high-quality conformational ensemble can improve agreement with experiment. However, to maintain consistency between parameter development and application for high-throughput tasks, we used a single conformation per molecule for parameter fitting and for all subsequent PBSA calculations. This strategy also avoids time-consuming conformational sampling steps and improves the computational throughput. Notably, TI calculations on the MNSolv data set were also performed during development of the ABCG2 charge model and achieved better accuracy, with an RMSE of 0.89 kcal/mol for SFE calculations.

**Table 1:** Error Metrics of Calculated Solvation Free Energy by Using PBSA and SMD with Respect to Experimental Measurements^a^

					PBSA^a^			B3LYP/6-31G*		
Solvent	*N*	ε	λ	*b*	MSE	MUE	RMSE	MSE	MUE	RMSE
*n*-Pentane	26	1.8371	–0.01990	3.4	0.03	0.44	0.64	–0.09	0.29	0.37
*n*-Hexane	59	1.8819	–0.01590	2.2	0.30	0.89	1.11	0.08	0.51	0.74
Heptane	69	1.9113	–0.01508	2.0	0.53	1.04	1.40	0.33	0.54	0.89
2,2,4-Trimethylpentane	32	1.9358	–0.02349	4.2	0.11	0.53	0.63	–1.68	1.68	1.73
*n*-Octane	38	1.9406	–0.02292	4.5	0.10	0.43	0.60	0.20	0.40	0.50
*n*-Nonane	26	1.9605	–0.02428	5.0	–0.01	0.34	0.51	0.21	0.35	0.44
*n*-Decane	39	1.9846	–0.02297	4.5	0.15	0.68	0.92	0.16	0.41	0.50
Cyclohexane	83	2.0165	–0.02897	6.0	0.47	0.88	1.11	0.44	0.63	0.81
*n*-Hexadecane	196	2.0402	–0.02081	3.7	0.25	0.95	1.23	0.41	0.68	0.97
Decalin-mixture	27	2.1960	–0.01319	1.8	0.42	0.85	1.06	0.65	0.73	0.89
Carbon tetrachloride	78	2.2280	–0.01852	2.8	0.29	0.82	1.05	0.13	0.49	0.68
Benzene	74	2.2706	–0.01743	2.3	0.16	0.80	1.07	0.68	0.88	1.19
Xylene-mixture	47	2.3879	–0.02269	4.2	0.12	0.51	0.74	0.62	0.69	0.81
Ethylbenzene	28	2.4339	–0.02573	5.2	0.11	0.50	0.77	0.41	0.49	0.63
Diisopropylether	21	3.3800	–0.03030	6.0	–0.07	0.81	1.00	0.52	0.82	1.14
Diethyl ether	71	4.2400	–0.03070	6.8	0.20	1.01	1.29	0.39	0.86	1.23
Iodobenzene	20	4.5470	–0.02796	6.7	0.01	0.62	0.76	–0.04	0.32	0.44
Chloroform	107	4.7113	–0.02258	4.7	0.30	1.02	1.40	0.45	0.89	1.20
Butyl acetate	21	4.9941	–0.02313	5.0	0.27	0.97	1.39	–1.08	1.31	1.60
Bromobenzene	27	5.3954	–0.02673	6.6	0.09	0.68	0.98	–0.28	0.41	0.50
Chlorobenzene	38	5.6968	–0.02874	7.2	0.09	0.57	0.80	–0.40	0.50	0.61
Ethyl acetate	23	5.9867	–0.02577	5.6	–0.03	0.85	1.14	–0.55	1.37	1.67
1,2-Dichloroethane	39	10.1250	–0.02376	6.7	–0.02	0.58	0.85	–0.05	0.58	0.87
1-Pentanol	22	15.1300	–0.03024	7.1	–0.19	0.74	1.04	0.27	0.94	1.21
1-Butanol	21	17.3323	–0.02943	7.2	–0.15	0.83	1.08	–0.05	0.98	1.31
Acetonitrile	7	35.6881	–0.02808	7.0	–0.02	0.57	0.72	–0.20	0.49	0.61
Dimethyl sulfoxide	7	46.8260	–0.02480	6.6	–0.04	0.93	1.03	0.81	0.98	1.07
**All**	**1246**				**0.13**	**0.73**	**1.10**	**0.09**	**0.71**	**0.98**

a *N* is the number of solutes associated with the solvents. ε is the dielectric constant. λ and *b* are solvent-specific parameters. MSE: mean signed error. MUE: mean unsigned error. RMSE: root-mean-square error. The units of MSE, MUE and RMSE are kcal/mol.

b Solvent-specific PBSA parameters for each solvent were listed according to eq 4.

### Partition Coefficients Calculations

Table 2 summarizes the RMSE, MAE, and MUE of logP calculated by PBSA, SMD, and TI relative to experiment measurements. In this section, all error metrics are reported in log units. The calculated logP values for all solutes are provided in the . Across 249 solutes spanning five water-organic solvent systems, PBSA yielded an overall RMSE of 2.02 log units, whereas SMD and TI achieved RMSEs of 1.55 and 1.59 log units, respectively. Overall, TI and SMD exhibited comparable accuracy for transfer free energies (and thus logP), while PBSA showed an approximately 0.5 log units larger RMSE.

**Table 2:** Error Metrics of Calculated logP using PBSA, SMD and TI on Test Set Water-Organic logP Values^a^

		PBSA			B3LYP/6-31G*			TI			PB+SMD_nonpolar		
Solvent^a^	*N*	MSE	MUE	RMSE	MSE	MUE	RMSE	MSE	MUE	RMSE	MSE	MUE	RMSE
Benzene	75	–0.09	1.20	1.59	–0.89	1.36	1.90	0.26	1.03	1.54	2.10	1.71	2.71
1-Butanol	41	2.53	2.57	3.10	0.57	1.14	1.50	0.52	1.68	2.37	1.30	1.15	1.52
Carbon tetrachloride	45	–0.18	1.28	1.69	–0.56	1.22	1.76	–0.21	1.15	1.52	1.91	1.45	2.57
1,2-Dichloroethane	68	–0.76	1.73	1.89	0.09	0.67	0.96	0.29	0.61	0.93	1.48	1.17	2.13
1-Pentanol	19	1.48	1.55	1.81	–0.52	0.90	1.22	–0.84	1.40	1.75	0.78	0.27	0.92
**All**	248	**0.60**	**1.67**	**2.02**	**–0.26**	**1.06**	**1.55**	**0.00**	**1.18**	**1.59**	**1.51**	**1.15**	**2.25**

a RMSE: root-mean-square error. MSE: mean signed error. MUE: mean unsigned error. The units of RMSE, MSE and MUE are log units.

b Only organic solvents are listed for representing the solvent systems.

At the solvent system level, PBSA performed better than SMD for the water–benzene (RMSE_PBSA_ = 1.59 and RMSE_SMD_ = 1.90) and water–carbon tetrachloride (RMSE_PBSA_ = 1.69 and RMSE_SMD_ = 1.76) systems. The outlier for PBSA was water–butanol, for which PBSA produced an RMSE of 3.10, and elevated errors were also observed for TI in this system. In the water–butanol set, the largest compound-level deviations were observed for riboflavin (PBSA: 8.75; SMD: −4.41; TI: −2.32), dihexyl phthalate (PBSA: 5.80; SMD: 2.04; TI: 3.31), neamine (PBSA: 5.65; SMD: −4.14; TI: −6.64) and decabromodiphenyl ether (PBSA: 4.93; SMD: 2.98; TI: 8.93). After excluding the four solutes, the RMSE for water–butanol decreased to 2.46 for PBSA, 1.08 for SMD, and 1.57 for TI. For PBSA, we found systematic errors for amine- and hydroxyl-containing solutes. These trends persisted when using PCM energies combined with SASA-based nonpolar terms, suggesting that additional treatment is needed for these functional groups for SFE calculation in the solvent butanol.

Note that the RMSE values for logP prediction are 3.10 and 1.81 log units for 1-butanol and 1-pentanol, respectively. In our previous work,^29^ we reported an RMSE of 0.91 log units for logP predictions of a large set of drug-like molecules (the Martel data set^56^) for the water/1-octanol system. These results indicate that our PBPK models perform better for more hydrophobic solvents, consistent with the hydrophobicity ranking 1-octanol > 1-pentanol > 1-butanol. Interestingly, logP values computed using the combination of PB polar and SMD nonpolar energies yield substantially lower RMSE values for both 1-butanol and 1-pentanol (Table 2). The marked improvement in prediction accuracy with the combined scheme (RMSE = 1.52 for 1-butanol and 0.92 log units for 1-pentanol) suggests that the scaled SAS approach used to estimate the nonpolar component of the solvation free energy requires refinement for more polar solvents. Future work will focus on developing more advanced methods to better model nonpolar contributions in polar solvent environments.

### Development and Testing of Solvation-Free-Energy-Based logBB Model

Some logBB predictive models were constructed by fitting linear regressions to physicochemical descriptors such as water/1-octanol logP, water/cyclohexane logP, solute surface area, and hydration free energy.^57–59^ Here, we developed an analogous linear model using PBSA-calculated SFEs in multiple solvents as descriptors. To preserve model simplicity and reduce the risk of overfitting, we limited the number of organic solvents to at most five. This model simulates the intrinsic transferability of molecules between different environments through combinations of solvents.

We then conducted an exhaustive search over all solvent combinations (174,436 in total) drawn from the 27 solvents parametrized in this study, together with 1-octanol, toluene, and cyclohexane from our previous work.^29,30^ HFE was always involved in multivariable linear regression. For each combination, we performed 10 bootstrap iterations to determine the optimal combination. In each iteration, the training set was randomly split into a 9:1 ratio with the smaller subset used for validation. One compound in the training set, methotrexate, was excluded from model fitting due to an anomalous HFE. The optimal model on the training set used SFEs in water (HFE), 1-octanol, carbon tetrachloride, *n*-pentane, *n*-octane, and xylene mixture as descriptors:

5 $$\mathrm{logBB}=-0.1116\times \mathrm{HFE}+4.5656\times SFE_{ocl}-1245.4075\times SFE_{car}-1170.7768\times SFE_{pen}+1506.9692\times SFE_{oce}+891.4166\times SFE_{xyl}-930.4952$$

where the HFE denotes the hydration free energy of solutes in water, and SFE denotes the solvation free energy of solutes in an organic solvent. Subscripts indicate the solvent: ocl, 1-octanol; car, carbon tetrachloride; pen, *n*-pentane; oce, *n*-octane; and xyl, xylene mixture. The optimal model was selected based on the average root-mean-square error across 10 bootstrap iterations. Subsequently, the coefficient of each SFE was fitted using the full training set.

The performance of the logBB predictive model is summarized in Table 3 and illustrated in Figure 2. The linear model shows a consistent accuracy for the training and test sets. On the training set, it achieves an RMSE of 0.65 and an MAE of 0.49. On the test set, the RMSE remains 0.65 and the MAE is 0.50. In addition, Kendall’s Tau increases to 0.45 on the test set relative to the training set, indicating improved rank ordering performance. Negative coefficients for water, carbon tetrachloride, and pentane reflect penalties associated with excessive hydrophilicity or nonproductive hydrophobic trapping, whereas positive coefficients for octanol, octane, and xylene indicate that favorable solvation in membrane-like and aromatic environments promotes BBB penetration. Overall, these results suggest good transferability across chemically diverse compounds with a stable predictive performance. For the classification task, a logBB threshold of −1.0 was applied to determine whether molecules can cross the BBB. Using this criterion, the model achieved an accuracy of 0.84, with a sensitivity of 0.98, and a specificity of 0.07. Considering the complexity of transmembrane transport mechanisms, it is important to evaluate the performance of our model on compounds involved in transport processes beyond passive diffusion, particularly those affected by active transport. We identified 38 molecules as P-glycoprotein (P-gp) substrates by querying the ChEMBL data set, of which 7 were included in the test set. The qualitative prediction accuracy for these 7 compounds reached 0.86, with only one molecule incorrectly predicted as capable of crossing the blood–brain barrier. These results suggest that the current model performance is not significantly biased by the presence of P-gp substrates.

**Table 3:** Error Metrics of Calculated logBB Values^a^

	*N*	MSE	RMSE	*R*	*R* ^2^	Tau
Train	865	0.49	0.65	0.55	0.30	0.41
Test	97	0.50	0.65	0.55	0.27	0.45

a N: number of molecules. RMSE: root-mean-square error.; MSE: mean signed error. *R*: Pearson’s correlation coefficient. *R* ^2^: coefficient of determination. Tau: Kendall’s Tau value. The units of all error metrics are log units.

**Figure 2:**
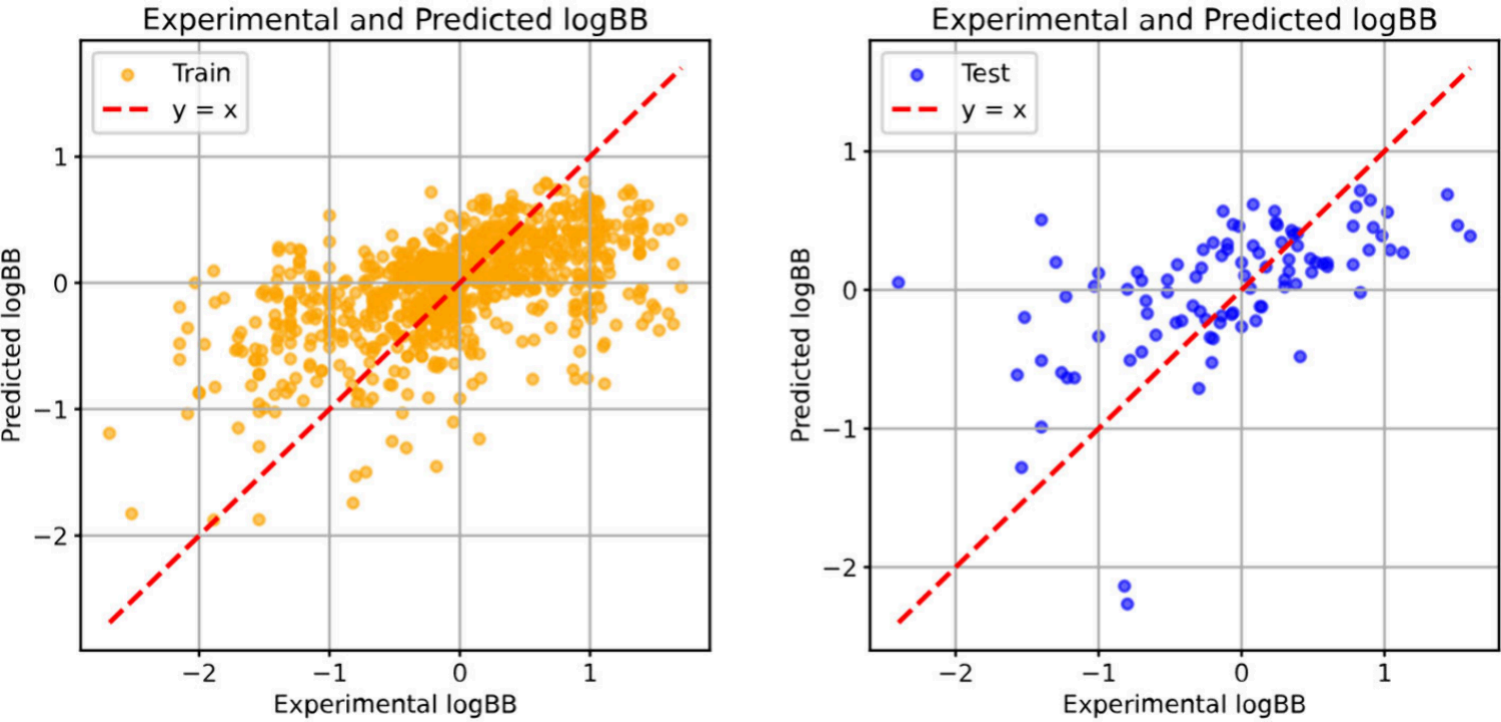
Correlation plots between experimental and predicted logBB for the training set (left) and the test set (right).

We finally examined two representative molecules from the test set: CHEMBL441965 (experimental logBB = −1.54, predicted logBB = −1.28) and butanediol divinyl ether (experimental logBB = 0.12, predicted logBB = 0.27). CHEMBL441965 contains four sp^3^ nitrogen atoms, which increase its capacity to form hydrogen bonds with water and are therefore consistent with low BBB permeability. In contrast, butanediol divinyl ether is a relatively hydrophobic, linear ether, which is more consistent with favorable BBB penetration. Notably, neither compound is reported as a substrate of P-gp.

## Conclusion

In this study, we parametrized the nonpolar solvent-accessible surface area (SASA) term for 27 organic solvents within a PBSA framework, using experimental solvation free energies from the MNSol database as target. These parameters enable PBSA to compute solvation free energies across diverse solvent environments, thereby extending its applicability to drug discovery workflows. The resulting PBSA model achieves accuracy comparable to that of quantum mechanical SMD for solvation free energies while offering significantly higher computational efficiency. We further demonstrate two applications of the model: predicting partition coefficients (logP) and blood–brain barrier permeability (logBB), two key physicochemical descriptors widely used in ADMET modeling.

## Supplementary Material





## Data Availability

PB energy was calculated using Delphi V4 (https://honig.c2b2.columbia.edu/delphi). Atom type and atomic charge were assigned by using Antechamber in AmberTools, which could be downloaded from the AMBER webpage (https://ambermd.org/AmberTools.php). Quantum mechanical calculations were performed using Gaussian 16 software. Molecular dynamics simulations were performed using pmemd.MPI and pmemd.cuda in AMBER 22 release. Data analysis was performed using Python and Scikit-learn package.

## Notes

PBSA and SMD calculated SFEs accompanied by solutes’ experimental measurements (CSV)
PBSA, SMD and TI calculated logP accompanied by solutes’ experimental logP (CSV)
